# Phosphodiesterase type 5 inhibitors do not prevent curvature progression but shorten pain duration in the active phase of Peyronie’s Disease: A retrospective cohort study

**DOI:** 10.1038/s41443-023-00810-5

**Published:** 2024-01-16

**Authors:** Emil Durukan, Rune Kraglund, Shad Azad Rashid, Tine Thorkilgaard, Christian Fuglesang Skjødt Jensen, Mikkel Fode

**Affiliations:** 1 Department of Urology, Copenhagen University Hospital, Herlev and Gentofte Hospital, Copenhagen, Denmark; 2 Department of Clinical Medicine, University of Copenhagen, Copenhagen, Denmark

**Keywords:** Sexual dysfunction, Medical research

## Abstract

Treatment with Phosphodiesterase Type 5 inhibitors (PDE5is) has shown promise in managing Peyronie’s disease (PD) during its active phase. In a retrospective cohort study of 133 PD patients, we compared daily PDE5i treatment (sildenafil 25 mg or tadalafil 5 mg) in Group 1 (n = 101) to no treatment in Group 2 (n = 32). The mean age ± SD was 58.5 ± 10, (range: 29-77) years in Group 1 and 59 ± 13.7 years (range: 23-80) in Group 2 (p = 0.5). Mean symptom onset-to-visit time was 10.6 ± 7.2 months (range: 1-37) in Group 1 and 11 ± 6.3 months (range 3-27) in Group 2 (p = 0.5). Mean penile curvature change was +0.87° (95% CI: -1.8, 3.5) in Group 1 and +5.72° (95% CI: 1.4, 10) in Group 2 (p = 0.07) between first and last observations. Group 1 experienced shorter mean pain duration (9.1 ± 4.7 months, range: 2.5-24) than Group 2 (12.2 ± 6.5 months, range: 5-28) (p = 0.04). When controlling for baseline curvature and symptom onset-to-visit time, there were no differences between groups (-4.7, 95% CI: -10, 0.6) (p = 0.08). In conclusion, continuous PDE5i treatment did not affect PD curvature progression but showed a promising effect on pain.

## Introduction

Peyronie’s disease (PD) is an acquired connective tissue disorder affecting 0.4-20.3% of all men with a typical age of onset of 50-60 years [1, 2].

The condition spans two phases. In the first phase, one or more localized fibrotic plaques is formed in the tunica albuginea of the penis. This is often associated with painful erections and evolving deformity of the erect penis [3]. In the second phase, also known as the fibrotic or chronic phase, the curvature stabilizes, and the pain subsides in the majority of patients. After this point, only a small percentage of patients (3-13%) experience spontaneous improvement in their penile deformity [4–7].

Currently, there are no established medical treatments aimed at addressing the progression of curvature during the active phase in PD [8]. Experimental trials on rats have implied that continuous treatment with Phosphodiesterase Type 5 inhibitors (PDE5is) may be a viable treatment [9]. In continuation of this, a few studies have investigated the use of PDE5is for the treatment of acute PD in humans with generally encouraging results. A retrospective study of 65 men suggested that tadalafil 2.5 mg daily could reduce isolated septal plaques when compared to untreated controls [10]. A subsequent prospective study of 39 men showed that sildenafil 50 mg daily for 12 weeks reduced pain in PD patients compared to vitamin E 400 IU treatment but it did not infer significant changes in curvature [11]. Finally, a recently published observational retrospective study with 191 men found that tadalafil 5 mg taken daily was associated with a lower rate of curvature progression at 12 weeks compared to controls [12].

Based on the somewhat limited evidence, we have been administering a daily PDE5i to our patients with active-phase PD at Herlev and Gentofte Hospital in Denmark since 2017. The objective of this study is to retrospectively evaluate its impact on penile curvature.

## Subjects and methods

We performed a retrospective cohort study of patients with PD seen in the outpatient clinic of Herlev and Gentofte Hospital, Denmark between January 2017 and July 2021. The study was registered and approved by The Danish Data Protection Agency in accordance with Danish law (P-2022-106).

### Patient evaluation

Patients were identified by searching for the diagnostic code for PD in the electronic hospital records. As a standard at our center, all patients had been evaluated by a board-certified urologist. Patients in the active phase of PD, defined as those experiencing painful erections and/or a continued development of their penile curvature, were included if they had at least two recorded visits with curvature measurements. The extent of penile curvature and the duration between symptom onset and the first clinical visit were not exclusion criteria. Patients receiving collagenase clostridium histolyticum injections, patients with an hourglass formation of the penis, and patients with hinge deformity were excluded.

We registered the following data from each patient: Age, BMI, comorbidities (depression, diabetes, connective tissue disorder, cardiovascular disease), and erectile function (categorized as either normal or impaired based on subjective patient report).

Curvature measurements were obtained using self-photography of a natural erection, as outlined in the European Urology Guidelines [8]. These measurements were conducted following written instructions that were provided prior to the scheduled visit. The degree of penile curvature was objectively measured by using a protractor. The protractor was positioned at the absolute center of the penile shaft, and the angle of the curve was measured from the point where the center of the shaft intersects the angle and ending at the tip of the penis. If photographs were unavailable, the curvature details were extracted from the written chart. The following additional PD characteristics were registered: PDE5i treatment, time from onset of symptoms to first clinical visit and the presence of painful erections.

Patients were divided into two groups, namely those who had received daily PDE5is as part of their treatment (Group 1) and those who had not (Group 2). Group 1 patients were administered PDE5is in tablet form. The decision to either administer sildenafil or tadalafil was made by the consulting urologist in collaboration with the patient. The primary outcome was the change in penile curvature between visits in patients receiving daily PDE5i treatment compared to patients receiving no treatment. As a secondary endpoint we compared the pain development between Group 1 and 2. During the clinical visits, patients were specifically queried regarding their adherence to daily medication. Those patients who demonstrated consistent medication intake and continued to remain in the active phase of the disease were retained as study participants. For patients who had terminated their daily medication, we recorded the cessation date. In these instances, the patient’s data were meticulously tracked, spanning from the initiation of medication to the moment of discontinuation. None of the patients had undergone treatment explicitly targeted PD such as Extracorporeal Shockwave Therapy (ESWT), vacuum therapy, or traction therapy. Patients who had previously been treated with on-demand PDE5is were not excluded. Patients who were originally designated to receive treatments apart from PDEis or concomitant on-demand treatment with PDE5is were not included in the study. In instances where patients initially initiated treatment with either PDE5is (Group 1) or opted for no treatment (Group 2) but were later redirected towards alternative treatment, we continued to monitor their penile curvature measurements until the initiation of the new treatment, which could include surgical intervention or collagenase clostridium histolyticum injections.

### Statistics

Statistical analysis was performed using the RStudio software version 2022.01.1. The test for normal distribution was performed using the Lilliefors test. The continuous variables are presented as means with standard deviations (SD) and categorical variables are presented as frequencies or percentages. The Wilcoxon rank-sum test was used to compare continuous variables while the Chi-squared test was used for comparing categorical variables. In relation to the secondary endpoint concerning the duration of pain, we also employed the Wilcoxon rank-sum test for analysis. In addition to analyzing mean differences in penile curvatures between first and last observations, we conducted a multiple regression analysis to adjust for baseline curvatures and the number of days between the onset of symptoms and the first clinical visit. The level of statistical significance was predetermined at p < 0.05.

## Results

A total of 133 patients were eligible for inclusion in the study. 101 patients (Group 1) received PDE5is daily. 2 patients received tadalafil (5 mg) while the rest received sildenafil (25 mg). 32 patients did not receive treatment (group 2). The primary reason for not administering treatment was patient refusal based on the risk of side effects, the uncertain clinical benefit, and in cases where it is was contraindicated. The mean age ± SD of the patients was 58.5 ± 10 years (range: 29-77) in group 1 and 59 ± 13.7 (range: 23-80) in group 2 (p = 0.5). The mean time ± SD from onset of symptoms to first visit was 10.6 ± 7.2 months (range 1-37) in group 1 and 11 ± 6.3 months (range: 3-27) in group 2 (p = 0.5). Group 1 patients were treated for a mean ± SD period of 10.9 ± 6.8 months (range: 3-36) and were monitored for a mean ± SD duration of 11.3 ± 7 months (range: 3-36). Group 2 patients were monitored for a mean ± SD duration of 13.6 ± 7.1 months (range: 3-30) (p = 0.07). Table 1 provides an overview of the baseline demographic and clinical characteristics for both groups.

**Table 1:** Clinical characteristics of each group.

	Group 1	Group 2	P value
	(*n* = *101*)	(*n* = *32*)	
Age, years, mean (SD)	58.5 (10)	59 (13.7)	0.5
BMI kg/m^2^, mean (SD)	25.7 (3.3)	26.5 (3.2)	0.3
Baseline curvature, mean degrees (SD)	33.5 (16.6)	32.2 (18.6)	0.5
Time from onset of symptoms to 1st visit, months, mean (SD)	10.6 (7.2)	11 (6.3)	0.5
Time from 1st visit to last visit, months, mean (SD)	11.3 (7)	13.6 (7.1)	0.07
Treatment duration, months, mean (SD)	10.9 (6.8)	-	
Erectile dysfunction, n (%)	30 (29.7)	7 (21.9)	0.4
Diabetes, n (%)	13 (12.9)	1 (3.1)	0.1
CVD, n (%)	9 (8.9)	5 (15.6)	0.3
Depression, n (%)	10 (9.9)	2 (6.3)	0.5
Connective tissue disorder, n	1 (1)	1 (3.1)	0.4

*BMI* Body mass index, *CVD* Cardiovascular disease.

We applied the Wilcoxon rank-sum test to analyze continuous variables, while the chi-squared test was used for categorical variables.

In group 1, the mean change in curvature progression from the first to the final clinical visit was +0.87 degrees (95% CI: -1.8, 3.5), while in group 2 it was +5.72 degrees (95% CI: 1.4, 10) (p = 0.07). Table 2 shows the proportions of patients in each group who experienced improvement, no change, or worsening in their curvatures.

**Table 2:** The number of patients exhibiting improvement, no change, or worsening in their curvature varied between the first and last observations.

	Group 1	Group 2	P value
Improvement, n (%)	31 (30.7)	6 (18.8)	0.2
No change, n (%)	39 (38.6)	11 (34.4)	0.7
Worsening, n (%)	31 (30.7)	15 (46.9)	0.09

The chi-square statistic is 3.2.

Among patients showing improvements or no change, the mean change in curvature progression was -5.59 degrees (95% CI: -7.7, -3.5) in group 1 and -5 (95% CI: -10, -0.04) in group 2 (p = 1). In group 1, 22 patients (21.8%) demonstrated an improvement of more than 10 degrees, compared to 3 patients (9.4%) in group 2. In Group 1, 69.3% exhibited either no change or an improvement in curvature progression. This percentage was higher in Group 2, where 81.3% demonstrated no change or improvement (p = 0.2). The multiple regression model did not reveal any statistically significant effect of PDE5is on changes in curvature when adjusting for baseline curvatures and time from symptom onset (p = 0.08) (Table 3). Nevertheless, our findings indicate that greater baseline curvatures were associated with a significant reduction in the change of curvature between the first and last observations (p = 0.02). For each 1-degree increase in the baseline curvature, the progression in curvature change would decrease by -0.17. This implies that the baseline curvature during the first visit significantly influences the subsequent change in curvature.

**Table 3:** Multiple regression model on the association between curvature change between first and last observation in each group adjusting for baseline curvatures and time from onset of symptoms to first visit.

Variable	Regression coefficient	Std. error	95% CI regression coefficients	P value
PDE5i Treatment	−4.7	2.7	−10 — 0.6	0.08
No PDE5i treatment	11.6	3.4	4.9 — 18.3	<0.01
Baseline curvature	−0.17	0.07	−0.3 — −0.03	0.02
Time from onset of symptoms to first visit	−0.001	0.003	−0.008 — 0.006	0.8

Mutliple regression analysis.

*PDE5i* Phosphodiesterase type 5 inhibitor.

In the analysis of our secondary endpoint, we observed a significantly shorter duration of pain in group 1 compared to group 2. Group 1 had a mean ± SD duration of pain for 9.1 ± 4.7 months (range: 2.5-24) while group 2 had a mean ± SD duration of pain for 12.2 ± 6.5 months (range: 5-28) (p = 0.04).

## Discussion

The use of PDE5is as a therapeutic option for the management of PD has garnered attention in recent years, yet the existing body of evidence on their efficacy is limited [12]. In this study, we sought to evaluate the effect of daily use of PDE5is in patients with PD in the active phase. In relative terms, we found a difference in development of the penile curvature between the two groups, which may be noteworthy as the change was +0.87° in the treatment group and +5.72° in the no treatment group. However, the difference did not reach statistical significance and is small compared to what may be considered clinically meaningful. Meanwhile, patients who received a daily dose of PDE5is had a shorter duration of pain compared to patient who did not receive treatment.

Experimental trials on rats have suggested that continuous administration with PDE5is may reduce plaque development in PD. In a study from 2003, Valente et al. demonstrated that long-term administration of the PDE inhibitors, sildenafil and pentoxifylline, and the nitric oxide synthases substrate, L-arginine, reduced collagen deposition and possibly increased apoptosis of fibroblasts and myofibroblasts, thereby inhibiting fibrotic plaques in a rat model of PD. The proposed mechanism was downregulation of transforming growth factor beta 1 (TGFb1)-induced [13]. Similarly in 2006, Ferrini et al. showed that long-term administration of vardenafil prevented the development of fibrotic plaques in a rat model of PD. The authors hypothesized that increased levels of cGMP induced by PDE-5 inhibition acted as an antifibrotic defense mechanism. Although continuous oral administration was less effective once the plaque was formed the study did demonstrate a moderate reduction in the size of a pre-formed plaque [9].

The results from experimental trials in rats have prompted further investigation into the impact of PDE5is on humans with PD. Most recently Spirito et al published a study similar to ours in which they evaluated the efficacy of daily tadalafil 5 mg in patients with PD in the acute phase [12]. The study compared 108 men receiving tadalafil and 83 who refused this treatment and found that tadalafil significantly reduced penile curvature progression at 12 weeks (25.9% of patients in the tadalafil group vs. 39.7% in the control group, p = 0.042). These findings have already made it into the European Urological Guidelines as an argument for conservative PD treatment with PDE5-inhibitors [8]. Meanwhile, the Spirito study and our study share several drawbacks warranting careful interpretation. Thus, they are both retrospective observational studies without randomization and possible underlying differences between patient groups. Further there is no blinding of the assessors which could lead to an overinterpretation of possible treatment benefits. In this regard, it is important to note that Spirito et al.’s overall group curvature changes were small in absolute numbers at 1.8 degrees in the tadalafil group and 10.1 degrees in the control group and that no statistical comparisons were made between the groups in this regard. As our studies come to opposite conclusions regarding a possible benefit of PDE5is on curvature development the data may be regarded as inconclusive when they are interpreted together. Meanwhile, the conflicting findings may also arise from other differences including use of different PDE5i treatment. In this regard the pharmacokinetics of sildenafil and tadalafil are different as the latter have a longer half-life meaning that the medication stays in the tissue for longer [14, 15]. This could infer a greater effect. Further patients presented about 2 months later for their first visit in our study compared to the Spirito study. As patients presented at different time points after experiencing their initial PD symptoms, drawing a definitive conclusion might be challenging due to the influence of time on the progression of PD. In our study, we performed a multiple regression analysis accounting for the time interval between the onset of symptoms and the first clinical visit. Nevertheless, there was no significant impact on the curvature changes when comparing the two groups.

Only two previous clinical trials have investigated the use of PDE5is in PD. The first one was a retrospective trial suffering from similar drawbacks as described above [10]. Here, the authors investigated the efficacy of daily tadalafil 2.5 mg compared to observation in men who had ultrasonographic-confirmed isolated septal scars [10]. In the study, 35 men received treatment for 6 months while 30 men served as controls. Resolution of septal scars were recorded in 24 patients (69%) in the tadalafil group compared to three patients (10%) in the control group. The final study was a small randomized controlled trial from 2014 [11]. Here, 39 patients were divided into two groups, receiving either 400 IU vitamin E (group 1) or 50 mg sildenafil (group 2) daily for 12 weeks. In group 1 the palpable plaque size decreased in 6 patients (33.3%) and in 7 patients (33.3%) in group 2. They also demonstrated a decrease in pain symptoms after treatment in 9 patient (42.8%) in group 1 and 14 patients (66.6%) in group 2. There were no differences between the two groups in terms of penile curvature and plaque volume. The study is limited by the small sample size, lack of double blinding and the short duration of the study period.

As evident, the combined data from human trials is inconclusive regarding the possible effects of PDE5-inhibitors on curvature development, while there is an indication of a possible effect regarding pain. Meanwhile, PDE5is are generally safe and well-tolerated with a low risk of side effects [16]. Further, the treatment may infer a secondary benefit as they improve erectile function, which may present an independent advantage in PD patients since it optimizes the overall chance of a satisfactory sex life. Therefore the drugs remain an attractive possibility in PD patients. In future trials, the assessment of the efficacy of PDE5is on curvature progression in active PD should be conducted through a randomized, double-blinded design hereby eliminating potential bias in patient selection and confounding variables. Further drug combinations may be of value. As an example, Ilg et al. investigated the effect of simvastatin in combination with PDE5is (sildenafil, vardenafil, and tadalafil) or Selective Estrogen Receptor Modulators (SERMs) (tamoxifen and raloxifene) on the transformation of fibroblasts to myofibroblasts in a rat model [17]. Statins have previously been suggested to have antifibrotic effects in other organs. The results showed that simvastatin, PDE5is, and SERMs all demonstrated a concentration-dependent decrease in myofibroblast transformation. Simvastatin inhibited myofibroblast transformation in a synergistic fashion when combined with vardenafil [17].

Our results in this study are based on a thorough assessment of patients with PD in the acute phase exclusively and we did not exclude patients with comorbidities such as cardiovascular disease or those taking other medications. This strengthens the generalizability of our findings. Additionally, our study had the most extended observation period to date, with a mean follow-up period of approximately one year for all patients. This timeframe allowed for a more extensive examination of the progression of PD, whereas previous studies monitored patients for only 12 weeks or 6 months. The limitations of our study include its retrospective design and relatively small observation group. This may have limited the statistical power to detect a treatment effect regarding curvature development. However, as we found only small absolute differences a clinically significant effect of single treatment with a PDE5i seems unlikely. The validity of our assessment of the PD phase, based on written charts, may have been limited due to its reliance on patient-reported symptoms of painful erections and/or the perceived progression of penile curvature. This possible issue is highlighted by the limited curvature development in both groups after the initial visit. In some cases where patients reported concurrent erectile dysfunction alongside PD, the photographed erection may not have provided the optimal basis for evaluating penile curvature. Additionally, noncompliance with daily medication intake by patients is a possibility.

## Conclusion

Continuous PDE5i treatment did not have an influence on the progression of curvatures in PD in our study. Meanwhile, the treatment reduced the duration of pain compared to the no treatment control group. These findings indicate that daily PDE5i treatment may be of value in men with active phase PD. However, the overall quality of evidence is low, and results are conflicting. Thus, further studies are needed to clarify the actual effects.

## Data Availability

The datasets analyzed during the current study are available from the corresponding author on reasonable request.
